# Threshold of main pancreatic duct for malignancy in intraductal papillary mucinous neoplasm at head-neck and body-tail

**DOI:** 10.1186/s12876-022-02577-3

**Published:** 2022-11-19

**Authors:** Hao Zhou, Xiaoshuang Li, Yajie Wang, Zhiyue Wang, Jingrong Zhu, Zhongqiu Wang, Xiao Chen

**Affiliations:** 1 Department of Radiology, Affiliated Hospital of Nanjing University of Chinese Medicine, Nanjing, 210029 China; 2 Department of Radiology, The Second Affiliated Hospital of Nanjing Medical University, Nanjing, 210000 China

**Keywords:** Intraductal papillary mucinous neoplasms, Main pancreatic duct, Malignancy, Dysplasia, Invasive carcinoma

## Abstract

**Background:**

Main pancreatic duct (MPD) dilation is a high-risk stigmata/worrisome feature of malignancy in intraductal papillary mucinous neoplasms (IPMNs). The threshold of MPD diameter in predicting malignancy may be related to the lesion location. This study aimed to separately identify the thresholds of MPD for malignancy of IPMNs separately for the head-neck and body-tail.

**Materials and methods:**

A total of 185 patients with pathologically confirmed IPMNs were included. Patient demographic information, clinical data, and pathological features were obtained from the medical records. Those IPMNs with high-grade dysplasia or with associated invasive carcinoma were considered as malignant tumor. Radiological data including lesion location, tumor size, diameter of the MPD, mural nodule, and IPMN types (main duct, MD; branch duct, BD; and mixed type, MT), were collected on computed tomography or magnetic resonance imaging. Serum carbohydrate antigen 19-9 levels, serum carcinoembryonic antigen levels, and the medical history of diabetes mellitus, chronic cholecystitis, and pancreatitis were also collected.

**Results:**

Malignant IPMNs were detected in 31.6% of 117 patients with lesions in the pancreatic head-neck and 20.9% of 67 patients with lesions in the pancreatic body-tail. In MPD-involved IPMNs, malignancy was observed in 54.1% of patients with lesions in the pancreatic head-neck and 30.8% of patients with lesions in the pancreatic body-tail (*p* < 0.05). The cutoff value of MPD diameter for malignancy was 6.5 mm for lesions in the head-neck and 7.7 mm for lesions in the body-tail in all type of IPMNs. In MPD-involved IPMNs, the threshold was 8.2 mm for lesion in pancreatic head-neck and 7.7 mm for lesions in the body-tail. Multivariate analysis confirmed that MPD diameter ≥ 6.5 mm (pancreatic head-neck) and MPD diameter ≥ 7.7 mm (pancreatic body-tail) were independent predictors of malignancy (*p* < 0.05). Similar results were observed in MPD-involved IPMNs using 8.2 mm as a threshold.

**Conclusion:**

The thresholds of the dilated MPD may be associated with IPMNs locations. Thresholds of 6.5 mm for lesions in the head-neck and 7.7 mm for lesions in the body-tail were observed. For MPD-involved IPMNs alone, threshold for lesions in the head-neck was close to that in the body-tail.

**Supplementary Information:**

The online version contains supplementary material available at 10.1186/s12876-022-02577-3.

## Introduction

Intraductal papillary mucinous neoplasms (IPMNs) are mucin-producing cystic tumors with a variable degree of dysplasia and are considered precursors of pancreatic ductal adenocarcinoma. According to the revised Fukuoka consensus guidelines [1], IPMN is subdivided into three types considering the degree of involvement of the pancreatic ductal system: main duct (MD) type, branch duct (BD) type, and mixed type (features of MD and BD, MT). IPMNs with MPD diameters not less than 10 mm, and/or with an enhanced mural nodule ≥ 5 mm are considered as high-risk stigmas and should be resected immediately [1]. In contrast, a diameter of MPD of 5–9 mm, cystic structure not less than 30 mm and an elevated serum carbohydrate antigen (CA) 19-9 level are considered worrisome features, suggesting nonoperative watchful management.

Nevertheless, several studies have challenged the cutoff value of MPD ≥ 10 mm as a high-risk stigma. Abdeljawad et al. [2] considered that a cutoff of 8 mm of MPD was able to discriminate benign from malignant MD-IPMNs. Hackert et al. [3] found that the malignancy risk was 59% in IPMNs with MPD sizes between 5 and 9 mm. Del Chiaro reported that a cutoff of 5 to 7 mm of MPD diameter was the best predictor to discriminate between malignant and benign IPMNs [4]. Roch et al. [5] pointed out that diffuse MPD dilation of IPMNs was an independent predictor of the development of invasive carcinomas. Diffuse dilation reflects diffuse MPD involvement of the tumor or an obstructing tumor located in the pancreatic head. However, they were ambiguous about the exact MPD dilation diameter that predicts the malignancy of IPMNs according to the lesion’s location in the pancreas (head-neck versus body-tail).

Interestingly, a recent study suggested that the threshold for the MPD diameter was different for MPD-involved IPMNs located in the pancreatic head-neck (9.0 mm) and body-tail (7.0 mm) [6]. However, this is the only study so far showing that the anatomic site of the gland should be considered when calculating the threshold. The validity of these results should be confirmed by other studies. Moreover, this study only investigated the MD/MT type of IPMNs. The thresholds are still unclear if BD-IPMNs were also included. Therefore, the aim of this study was to identify the threshold of MPD for identifying malignancy of IPMNs considering the tumor location (head-neck versus body-tail).

## Materials and methods

### Patients

This retrospective study was approved by the Institutional Ethics Review Board of the Affiliated Hospital of Nanjing University of Chinese Medicine. All procedures in this study adhered to Declaration of Helsinki. This study included 185 patients with pathologically proven IPMNs who underwent surgery during 2011–2021. Any patients with missing data were excluded. Surgery was performed based on Fukuoka guidelines, or because of obvious clinical symptoms and patient’s request. Patient demographic information, clinical data, and pathological features were obtained from the medical records. Fasting plasma glucose levels and 2-h plasma glucose levels were obtained within one week before the operation. Diabetes mellitus (DM) was defined according to the plasma glucose levels and a history of DM. We collected data of preoperative symptoms (such as abdominal symptoms and overt jaundice), serum carbohydrate antigen 19-9 (CA19-9) levels and serum carcinoembryonic antigen (CEA) levels. Medical histories of chronic cholecystitis and pancreatitis were also collected. Imaging information was obtained from the Picture Archiving and Communication System.

### Imaging data

The following radiological data were collected on computed tomography (CT) or magnetic resonance imaging (MRI): lesion location (head and neck vs. body and tail), tumor sizes, diameter of the main pancreatic duct (MPD), and mural nodule (enhanced solid component with a size ≥ 5.0 mm). The MPD diameter was measured at the site of the maximal dilation of the pancreatic duct on the Picture Archiving and Communication System. If the lesion was too large, the location was evaluated based on the site of the center of the cyst. If there were multiple lesions, the location was judged based on the main cyst. MD-IPMN was considered when segmental or diffuse involvement of the MPD was observed; BD-IPMN was considered when the lesions communicated with the MPD [7]. MT-IPMN was defined when the lesions had features of both MD- and BD-IPMNs. We combined MD-IPMN and MT-IPMN as MPD-involved IPMNs. The imaging features were reviewed blindly and independently by two radiologists (with extensive experience in pancreatic radiology) with no prior knowledge of the detailed histopathological information of any patients.

### Histological examinations

The histological diagnosis of IPMN was based on the World Health Organization guidelines for IPMNs. IPMNs were classified into low-intermediate dysplasia, high-grade dysplasia, and invasive adenocarcinoma. Malignant IPMNs were defined as those with high grade dysplasia or with associated invasive carcinoma. Lymph node metastasis (yes vs. no) and peripancreatic extension (organ invasion and vascular invasion) were also evaluated.

### Statistical analysis

The data analyses were performed with SPSS 20.0 (IBM Corp., Armonk, NY, USA). Continuous data are shown as the mean ± standard deviation and qualitative data are shown as numbers (percentage). Continuous data, such as patient age, tumor size, the serum levels of CEA and CA19-9, and MPD diameter were evaluated by independent-sample t tests or Mann–Whitney U tests. Qualitative data, such as sex, dysplasia level, tumor type, tumor location, chronic cholecystitis, pancreatitis, abdominal symptoms, lymph node metastasis, peripancreatic extension, and mural nodules, were subsequently compared by the Chi-square test or Fisher’s exact test. A receiver operating curve was used to calculate the threshold of MPD in identifying malignant IPMNs. Univariable and multivariable logistic regression analyses were used to evaluate the association of MPD with invasive carcinoma and malignant IPMNs. Logistic regression analyses were also performed to show the association of MPD with invasive carcinoma and malignant IPMNs in MPD-involved IPMNs. *P* values less than 0.05 were considered as statistically significant. The interobserver agreements were calculated using the weighted kappa values as follows: 0.00–0.20, poor agreement; 0.21–0.40, fair agreement; 0.41–0.60, moderate agreement; 0.61–0.80, good agreement; and 0.81–1.00, excellent agreement.

## Results

### Clinicopathological features of IPMNs

In our study, a total of 185 patients were included and their clinical data are shown in Table 1. There were 134 patients who had low-intermediate grade neoplasms and 51 patients who had tumors with high-grade or carcinoma. Low-intermediate grade IPMNs were more commonly seen in BD patients, while high-grade IPMNs and carcinoma were more commonly seen in patients with main type or/and mixed type IPMNs (*p* < 0.01). In addition, significant differences were found in serum CEA and CA19-9 levels (*p* < 0.01) between these two groups. Patients with malignant IPMNs had larger size of MPD diameters and mural nodules (*p* < 0.001). Extrapancreatic extensions were only seen in malignant IPMNs (*p* < 0.001). DM were more common seen in patients with malignant IPMNs than in those with low-intermediate grade IPMNs. However, no significant differences were found in patient age, sex, tumor size, tumor location, pancreatitis, abdominal symptoms or lymph node metastasis between the patients with and without malignant IPMNs.

**Table 1:** Clinicopathological features of intraductal papillary mucinous neoplasms (IPMNs)

	Total (n = 185)	Low-intermediate grade (n = 134)	High-grade/invasive carcinoma (n = 51)	*p*
Age (years)	63.15 ± 9.34	63.13 ± 9.12	63.18 ± 10.00	0.481
Size (cm)	3.65 ± 2.10	3.51 ± 2.17	4.02 ± 1.86	0.170
Sex (male/female)	112/73	77/57	35/16	0.165
*Type*				
Main	24 (12.97%)	9 (6.71%)	15 (29.41%)	< 0.001
Branch	97 (52.43%)	87 (64.92%)	10 (19.61%)	
Mixed	64 (34.59%)	38 (28.36%)	26 (50.98%)	
*Type2*				
Main and mixed	88 (47.57%)	47 (35.07%)	41 (80.39%)	< 0.001
Branch	97 (52.43%)	87 (64.92%)	10 (19.61%)	
*Location*				
Head and neck	118 (63.78%)	80 (59.70%)	37 (72.54%)	0.118
Body and tail	67 (36.22%)	53 (40.30%)	14(27.45%)	
CEA (ng/ml)	3.43 ± 3.36	2.96 ± 1.83	4.69 ± 5.51	< 0.001
CA19-9 (U/ml)	53.06 ± 221.91	31.44 ± 97.69	110.55 ± 390.58	0.004
MPD diameter (mm)	5.91 ± 4.31	4.88 ± 3.65	8.60 ± 4.77	0.006
Mural nodule	23 (12.43%)	9 (6.72%)	14 (27.45%)	< 0.001
Lymph node metastasis (yes)	2 (1.08%)	0	2 (3.92%)	0.075
Extra-pancreas extension (yes)	6 (3.24%)	0	6 (11.76%)	< 0.001
Diabetes Mellitus (yes)	32 (17.29%)	17 (12.69%)	15 (29.41%)	0.007
Symptom (yes)	80 (43.24%)	55 (41.04%)	25 (49.02%)	0.348
Pancreatitis (yes)	4 (2.16%)	4 (2.99%)	0	0.577
Complications (yes)	67 (36.22%)	50 (37.31%)	17 (33.33%)	0.615

*CA19-9* carbohydrate antigen 19-9, *CEA* carcinoembryonic antigen, *MPD* main pancreatic duct

### Clinicopathological features of MPD-involved type IPMNs

Next, we analyzed the clinicopathological features of MPD-involved IPMNs with and without malignant characteristics (Table 2). No significant differences were found for age, sex, tumor size, serum CEA level, serum CA19-9 level, lymph node metastasis, abdominal symptoms or complications between patients with low-intermediate grade IPMNs and high-grade/invasive carcinomas. In contrast, in patients with MPD-involved IPMNs, more than half of patients (54.1%) with IPMNs in the pancreatic head and neck had malignant tumors, which was significantly higher than that in patients with IPMNs in the pancreatic body and tail (30.8%) (*p* < 0.05). A larger MPD diameter, the presence of mural nodules and DM, and extrapancreatic extension were more common seen in high-grade IPMNs/invasive carcinomas than in low-intermediate grade IPMNs (*p* < 0.05).

**Table 2:** Clinicopathological features of main pancreatic duct (MPD)-involved type intraductal papillary mucinous neoplasms (IPMNs)

	Total (n = 88)	Low-intermediate grade (n = 47)	High-grade/invasive carcinoma (n = 41)	*p*
Age (years)	64.52 ± 8.83	64.94 ± 8.29	64.05 ± 9.48	0.641
Size (cm)	3.88 ± 1.94	3.95 ± 2.09	3.79 ± 1.75	0.702
Sex (male/female)	55/33	27/20	28/13	0.294
*Location*				
Head and neck	61 (69.32%)	28 (59.57%)	33 (80.49%)	0.046
Body and tail	26 (30.68%)	18 (40.43%)	8 (19.51%)	
CEA (ng/ml)	4.08 ± 4.46	3.24 ± 2.22	5.03 ± 5.99	0.060
CA19-9 (U/ml)	66.93 ± 299.17	16.04 ± 25.78	126.74 ± 435.65	0.085
MPD diameter (mm)	9.03 ± 4.33	8.15 ± 4.29	10.01 ± 4.22	0.045
Mural nodule (yes)	14 (15.91%)	4 (8.51%)	10 (24.39%)	0.042
Lymph node metastasis (yes)	2 (2.27%)	0	2 (4.88%)	0.214
Extra-pancreas extension (yes)	5 (5.68%)	0	5 (12.20%)	0.019
Symptom (yes)	38 (43.18%)	17 (36.17%)	21 (52.22%)	0.155
Pancreatitis (yes)	0	0	0	–
Diabetes Mellitus (yes)	17 (17.29%)	4 (8.51%)	13 (31.70%)	0.006
Complications (yes)	37 (42.05%)	20 (42.55%)	17 (41.46%)	0.918

*CA19-9* carbohydrate antigen 19-9, *CEA* carcinoembryonic antigen

### Threshold of MPD in identifying malignancy in IPMNs of head-neck/body-tail

**Fig. 1:**
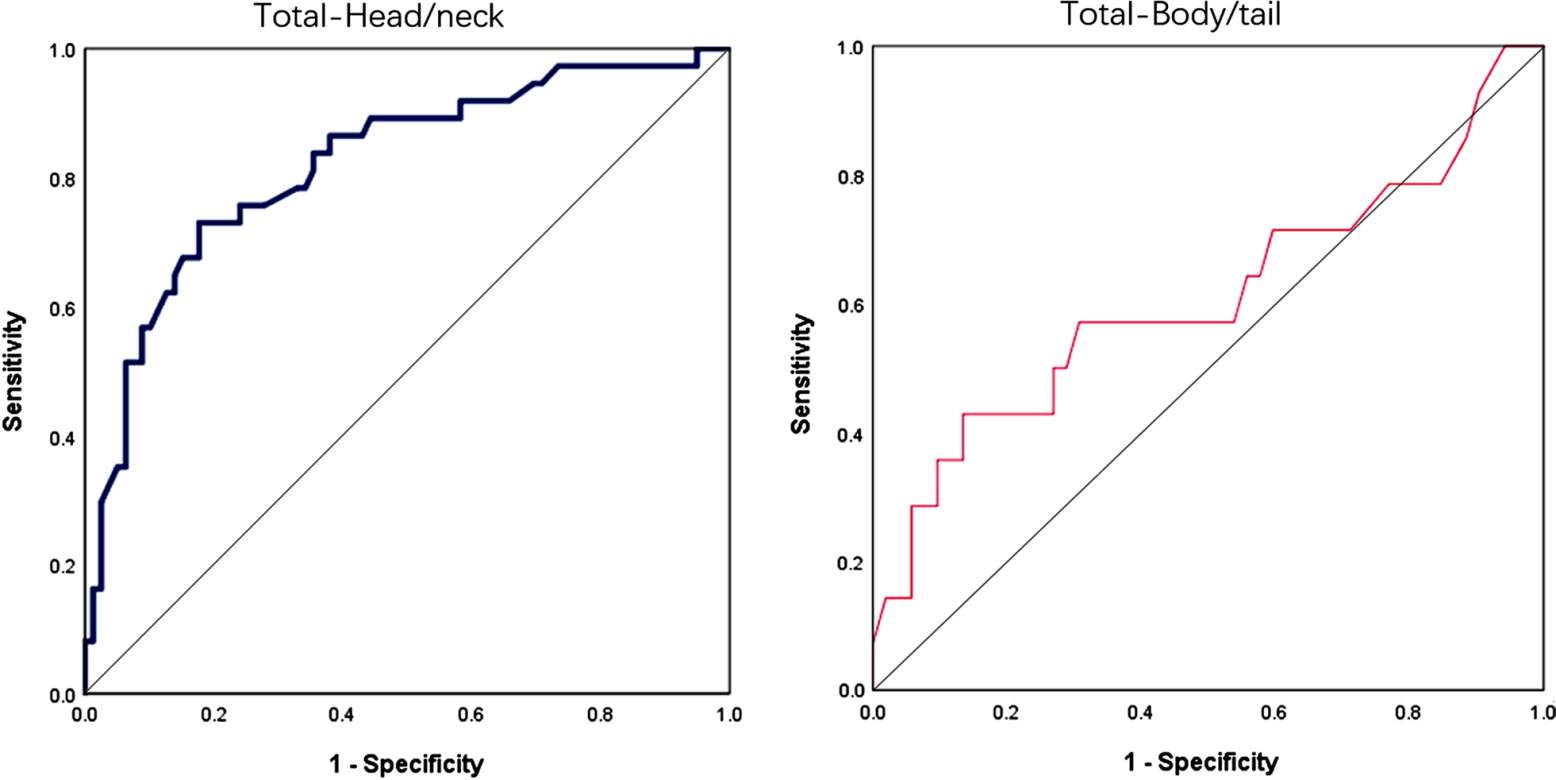
Receiver operating curve to calculate the threshold of main pancreatic duct (MPD) in identifying malignancy in all type intraductal papillary mucinous neoplasms (IPMNs). The threshold was 6.5 mm for lesions at head-neck and was 7.7 mm for lesions at body-tail

By analyzing the association between MPD diameter and the malignancy of IPMNs, we found that the cutoff value of the MPD diameter to distinguish benign from malignant IPMNs was 6.5 mm for head-neck IPMNs (area under the curve (AUC) = 0.82, sensitivity = 0.73, specificity = 0.82) and was 7.7 mm for body-tail IPMNs (AUC = 0.61, sensitivity = 0.43, specificity = 0.87), respectively (Fig. 1).

Univariable and multivariable logistic regression were used to show the association of the calculated threshold of MPD with malignant IPMNs (Tables 3 and 4). An MPD not less than 6.5 mm was associated with a higher risk of malignant IPMNs in univariate analysis (odds ratio (OR), 12.73; 95% CI 5.04–32.16, *p* < 0.001) and multivariate analysis (OR, 14.14; 95% CI 4.61–43.39, *p* < 0.001) for IPMNs at pancreatic head and neck (Table 3). For body-tail IPMNs (Table 4), MPD not less than 7.7 mm was an independent predictor of malignancy on univariate analysis (OR, 4.93; 95% CI 1.31–18.52, *p* = 0.018) and multivariate analysis (OR, 9.83; 95% CI 1.39–69.57, *p* = 0.022). CA19-9 levels not lower than 37 U/mL were also identified as predictors of malignancy for IPMNs of the head-neck (Table 3) and body-tail (Table 4) of the pancreas. The presence of mural nodules was associated with malignancy for IPMNs of the body-tail of the pancreas (Table 4). An MPD diameter of 6.5 mm was shown to have fair agreement (kappa = 0.52) with the pathological results in identifying malignancy.

**Table 3:** Association of calculated threshold of main pancreatic duct (MPD) with malignancy in head-neck intraductal papillary mucinous neoplasms (IPMNs)

	Univariate analysis		Multivariate analysis	
	OR	*P* value	OR	*P* value
MPD diameter (≥ 6.5 mm vs. < 6.5 mm)	12.73 (5.04–32.16)	< 0.001	14.14 (4.61–43.39)	< 0.01
Size (≥ 30 mm vs. < 30 mm)	3.63 (1.48–8.89)	0.005	2.39 (0.77–7.45)	0.13
CA19-9 (≥ 37 U/ml vs. < 37 U/ml)	3.30 (1.39–7.80)	0.006	3.68 (1.05–12.87)	0.04
Mural nodule (yes vs. no)	2.93 (1.03–8.36)	0.04	1.77 (0.45–7.00)	0.43
Diabetes Mellitus (yes vs. no)	5.07 (1.80–14.31)	0.002	2.01 (0.51–7.92)	0.32
CEA (≥ 5 vs. < 5 ng/ml)	4.02 (1.31–12.32)	0.015	1.23(0.23–8.68)	0.39

*CA19-9* carbohydrate antigen 19-9, *CEA* carcinoembryonic antigen, *OR* odds ratio

**Table 4:** Association of calculated threshold of main pancreatic duct (MPD) with malignancy in body-tail intraductal papillary mucinous neoplasms (IPMNs)

	Univariate analysis		Multivariate analysis	
	OR	*P* value	OR	*P* value
MPD diameter (≥ 7.7 mm vs. < 7.7 mm)	4.93 (1.31–18.52)	0.018	9.83 (1.39–69.57)	0.022
Size (≥ 30 mm vs. < 30 mm)	4.97 (1.01–24.39)	0.048	3.65 (0.40–33.31)	0.25
CA19-9 (≥ 37 U/ml vs. < 37 U/ml)	14.17 (2.37–84.54)	0.004	28.69 (2.50–329.06)	0.007
Mural nodule (yes vs. no)	28.89 (3.01–277.02)	0.004	50.42 (2.79–912.89)	0.008
Diabetes Mellitus (yes vs. no)	1.17 (0.28–5.01)	0.83	2.21 (0.32–15.56)	0.42
CEA (≥ 5 vs. < 5 ng/ml)	0.72 (0.14–3.72)	0.69	0.32 (0.03–4.01)	0.38

*CA19-9* carbohydrate antigen 19-9, *CEA* carcinoembryonic antigen, *OR* odds ratio

### Threshold of MPD in identifying malignancy in MPD-involved IPMNs of the pancreatic head-neck/body-tail

**Fig. 2:**
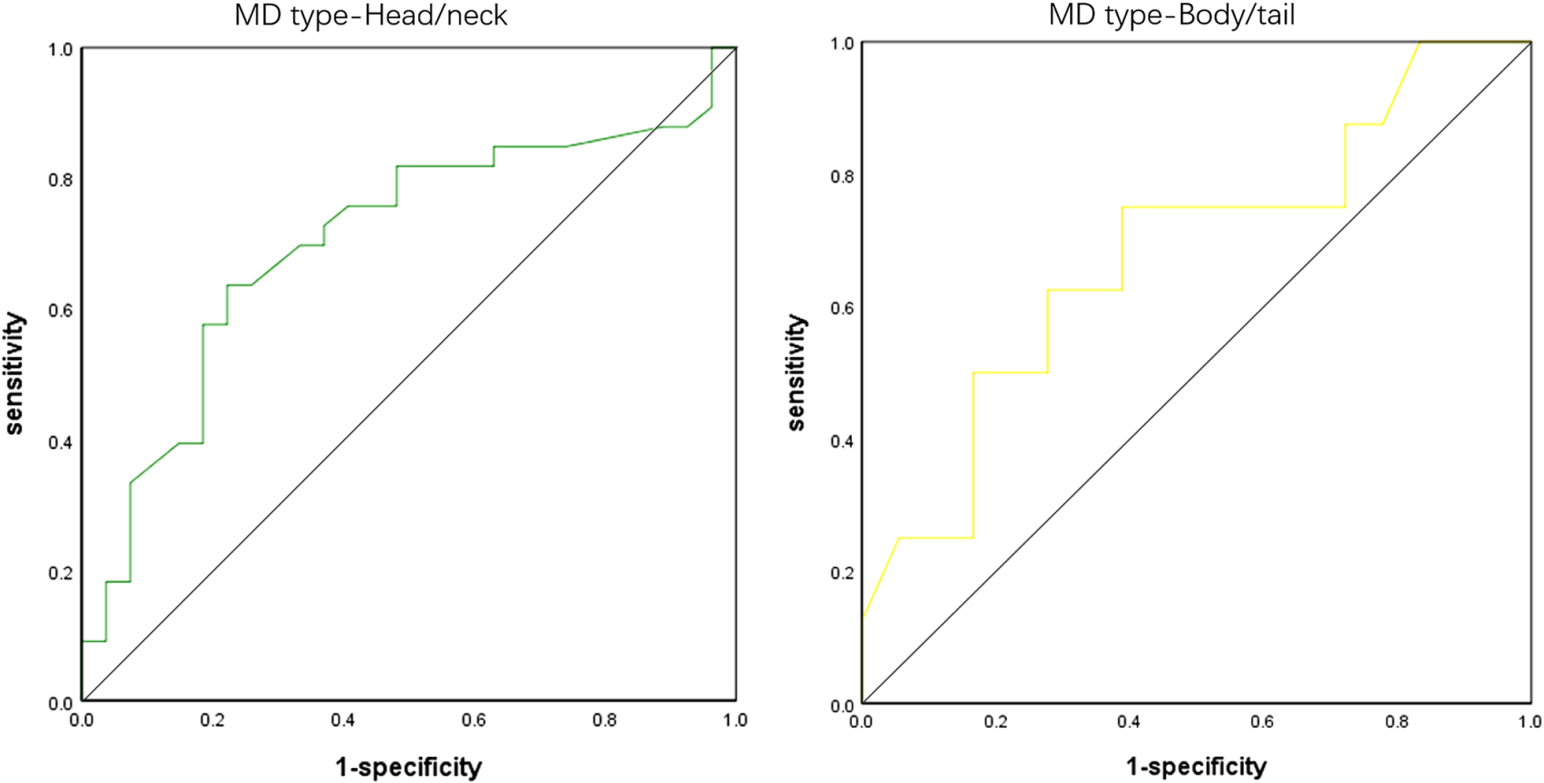
Receiver operating curve to calculate the threshold of main pancreatic duct (MPD) in identifying malignancy in MPD-involved intraductal papillary mucinous neoplasms (IPMNs). The threshold was 8.2 mm for lesions at head-neck and was 7.7 mm for lesions at body-tail

The best cutoff value of MPD diameter to distinguish between malignant and benign MPD-involved IPMNs with was 8.2 mm for head-neck IPMNs (AUC = 0.70, sensitivity = 0.64, specificity = 0.78) and was 7.7 mm for body-tail IPMNs (AUC = 0.68, sensitivity = 0.75, specificity = 0.61), respectively (Fig. 2). Subsequently, we evaluated the association between calculated threshold of MPD and malignant IPMNs in patients with MPD-involved IPMNs (Table 5). For head and neck IPMNs, an MPD diameter larger than 8.2 mm was associated with a higher risk of malignant IPMNs (OR = 9.75; 95%CI, 2.15–44.07). However, no significant correlation was observed between the calculated threshold and malignancy of IPMNs in pancreatic body and tail (*p* = 0.07) (Table 6). An MPD diameter of 8.2 mm was shown to have fair agreement (kappa = 0.42) with the pathological results in identifying head-neck malignancy. If using 6.5 mm as a threshold for malignancy in MPD-involved IPMNs located at head-neck, the kappa value was 0.31.

**Table 5:** The association between calculated threshold of main pancreatic duct (MPD) and malignancy in head-neck MPD-involved intraductal papillary mucinous neoplasms (IPMNs)

	Univariate analysis		Multivariate analysis	
	OR (95%CI)	*P* value	OR	*P* value
MPD diameter (≥ 8.2 mm vs. < 8.2 mm)	5.73 (1.86–17.63)	0.002	9.75 (2.15–44.07)	0.003
MPD diameter (≥ 7.7 mm vs. < 7.7 mm)	4.86 (1.64–14.39)	0.004	–	–
Size (≥ 30 mm vs. < 30 mm)	3.22 (1.05–9.84)	0.040	6.64 (1.43–30.88)	0.016
CA19-9 (≥ 37 ng/ml vs. < 37 ng/ml)	5.00 (1.42–17.64)	0.012	6.85 (1.14–40.83)	0.04
Mural nodule (yes vs. no)	2.67 (0.63–11.23)	0.18	1.30 (0.21–8.12)	0.78

*CA19-9* carbohydrate antigen 19-9, *OR* odds ratio

**Table 6:** The association between calculated threshold of main pancreatic duct (MPD) and malignancy in body-tail MPD-involved intraductal papillary mucinous neoplasms (IPMNs)

	Univariate analysis		Multivariate analysis	
	OR	*P* value	OR	*P* value
MPD diameter (≥ 7.7 mm vs. < 7.7 mm)	4.71 (0.73–30.28)	0.102	8.78 (0.84–91.49)	0.07
Size (≥ 30 mm vs. < 30 mm)	0.60 (0.08–4.54)	0.621	0.24 (0.02–3.42)	0.27
CEA (≥ 5 vs. < 5 ng/ml)	0.71 (0.06–8.15)	0.79	0.52(0.04–7.23)	0.62

*CA19-9* carbohydrate antigen 19-9, *CEA* carcinoembryonic antigen, *OR* odds ratio

Ca19-9 levels and presence of mural nodule were not included in the analysis because there were no cases with high CA19-9 (> 37 U/L) and presence of mural nodule in low-intermediate grade group. Then CEA was included in the models–

### Threshold of MPD in identifying malignancy in BD-IPMNs of the pancreatic head-neck//body-tail

The best cutoff value of MPD diameter to distinguish between malignant and benign BD-IPMNs was 2.9 mm for head-neck IPMNs (AUC = 0.66, sensitivity = 1.00, specificity = 0.38) (Additional file 1: Fig. S1A) and 3.1 mm for body-tail IPMNs (AUC = 0.64, sensitivity = 0.32, specificity = 1.00) (Additional file 1: Fig. S1B).

## Discussion

It is well-known that dilation of MPD is associated with malignancy in IPMNs. For IPMNs with MPD diameter ≥ 10 mm, surgery is recommended to prevent any possible progression to pancreatic cancer. However, a few studies have indicated that the threshold should be lower than 10 mm [8, 9]. Moreover, a study reported that the threshold was related to the tumor location for MD/MT IPMNs. Our present data also demonstrated that the threshold of MPD diameter was lower than 10 mm for identifying malignant IPMNs, 6.5 mm for lesions in pancreatic head-neck and 7.7 mm for lesions in body-tail. Our data also supported that the threshold of MPD diameter in identifying malignant MPD-involved IPMNs of pancreatic head-neck should be set at 8.0–9.0 mm.

Crippa et al. [6] stressed that MPD diameters for malignancy are different in MPD-involved IPMNs of the pancreatic head and body-tail. According to their study, surgery was recommended for MPD-involved IPMNs with MPD ≥ 9 mm in the pancreatic head and for IPMNs with MPD ≥ 7 mm in the pancreatic body-tail, respectively. In addition, for IPMNs with MPD < 8 mm in the head and MPD > 6 mm in the body-tail with high-risk stigma, immediate surgical resection was also suggested. However, this solitary research required validation by other studies. Moreover, BD-IPMNs were not included in that study. Our results showed that main-duct involved IPMNs with MPD ≥ 8.2 mm in the pancreatic head and with MPD ≥ 7.7 mm in the pancreatic body-tail were associated with malignancy of IPMNs, respectively. Our results were close to those reported by Crippa et al. Moreover, we also calculated the threshold of the MPD diameter for all types of IPMNs. The cutoff values of MPD diameter were 6.5 mm and 7.7 mm for malignancy of IPMNs in the pancreatic head-neck and body-tail, respectively. Our results showed that the threshold of the MPD diameter for distinguishing malignancy in the pancreatic head and neck was smaller than that reported by Crippa et al. They analyzed the size of the MPD diameter of MPD-involved IPMNs, but they did not consider BD-IPMNs. We took BD-IPMNs into account because the lesions located in the branch duct might communicate with the MPD and their aggressive behavior could influence the dilation of the MPD. Yoshioka et al. [10] showed that BD-IPMNs with MPD diameter more than 3 mm and a DM history had a higher risk of pancreatic ductal adenocarcinoma. Our data showed similar thresholds both in head-neck and body tail IPMNs. Interestingly, a recent study indicated that a cutoff of 5 to 7 mm MPD diameter was the best predictor to discriminate between malignant and benign IPMNs [4]. Ateeb et al. also indicated that main pancreatic duct dilation greater than 6 mm is associated with an increased risk of malignancy [11]. Our threshold of 6.5 mm is consistent with these findings. Moreover, the results of our study and those of Crippa et al. both supported that the threshold should be set at 7–8 mm for IPMNs located in the body-tail.

In addition, our data also showed that a higher risk of malignancy was found in IPMNs located in the pancreatic head and neck than those located in the pancreatic body and tail which consistent with the results of other studies. Kerlakian et al. [12] showed that lesions of the pancreatic head and uncinate were more likely to harbor malignancy than lesions of the body and tail. Jones et al. [13] indicated that IPMNs located in pancreatic head were associated with malignancy in univariate analysis. Suzuki et al. [14] pointed out that a tumor location in the pancreatic head was one of the predictive factors for malignant IPMNs. However, the reason was unclear just like the pancreatic ductal adenocarcinoma occurs more frequently in the pancreatic head. IPMNs located in the pancreatic head and neck might already have aggressive behaviors and be resected before a significant dilation of the MPD occurs.

In addition to the dilation of the MPD diameter, tumor size > 30 mm and CA19-9 level ≥ 37 U/mL were also found to be risk factors for malignancy of IPMNs in the pancreatic head and neck in the present study. Ciprani et al. [15] indicated that 63% of IPMN patients with a CA19-9 higher than 37U/mL were associated with malignancy and a poor prognosis. IPMNs located in the head and neck of the pancreas with these risk factors should be given more attention and surgical resection should be considered instead of surveillance. However, further study is needed to evaluate the association between these risk factors and malignant IPMNs in the pancreatic body and tail.

Hirono et al. [16] showed that IPMNs with lesions located in the pancreatic body/tail had a higher risk of recurrence in the remnant pancreas after surgery. They explained this phenomenon by neoplastic cells being transferred downstream through the flow of pancreatic juice to implant in the pancreatic duct epithelium. Lesions implanted in the pancreatic duct eventually led to dilation of the MPD. This finding might explain the difference in the MPD diameter in the pancreatic head-neck and body-tail in our study. Huang et al. [17] also stressed that being located in the pancreatic body/tail was an independent prognostic factor related to distant metastases of IPMNs. They illustrated that cancer-specific survival was shorter for head lesions than for body/tail lesions, which possibly contributed to a tumor progression.

In addition, MT-IPMNs in main-duct involved IPMNs in our study were more common seen than MD-IPMNs. This difference in tumor types might lead to a smaller MPD diameter in the present study. Some MT-IPMNs may have only minimal involvement of the MPD and no MPD dilation [18]. Therefore, the size of the MPD to discriminate benign and malignant IPMNs in MPD involved IPMNs in our study (8.2 mm) was slightly smaller than that in the previous study (9 mm).

Endoscopic ultrasound (EUS) and EUS-guided the needle biopsy are useful in the management and diagnosis of pancreatic solid [19] or cystic lesions [20–22]. Li et al. [23] reported that EUS with or without fine-needle aspiration had better performance in diagnosing pancreatic cystic neoplasms (PCNs) than CT or MRI. EUS also showed slight better performance in characterizing internal structures, such as septa and mural nodules [23]. A recent meta-analysis further showed that contrast-enhanced EUS (CE-EUS) had good ability for the characterization of mural nodules within PCNs [24]. However, EUS is not routinely performed for pancreatic diseases in our institution. Therefore, the imaging evaluations in our study were mainly based on the CT or MRI.

There are several limitations of our study. First, the number of MD-IPMNs was relatively small, and larger data sets are needed for a further study. Second, our study was a single-institution retrospective study which may cause selection bias, and a multicenter study should be performed to test our findings. Third, as a retrospective study, we did not evaluate the association between MPD diameter and survival or recurrence after surgery and a prospective study is necessary in the future. Fourth, the IPMN patients who did not undergo surgical resection were not included in our analysis, such as those who underwent surveillance. The loss of these populations may affect the results. Nevertheless, surgery may be performed for 40% of patients without "worser" features as their request or having clinical symptoms in our study. These subjects were complementary to the patients who underwent surveillance. Therefore, our results may also be generalizable for identifying malignancy in all IPMNs.

In conclusion, our study shows a slight difference in MPD thresholds for malignancy between IPMNs of the pancreatic head and body-tail in the MD/MT type. Moreover, a lower MPD threshold (6.5 mm) was observed for malignant IPMNs of the pancreatic head-neck for all types of IPMNs. For MPD-involved IPMNs alone, threshold for lesions in the head-neck was close to that in the body-tail (8.2 mm and 7.7 mm). Further investigations that focused on the association between MPD diameter and tumor location are needed to show the role of the MPD diameter in predicting the malignancy of IPMNs more accurately.

## Supplementary Information

**Additional file 1: Fig. S1**. Receiver operating curve to calculate the threshold of main pancreatic duct (MPD) in identifying malignancy in branch-duct (BD)-intraductal papillary mucinous neoplasms (IPMNs). The threshold was 2.9 mm for lesions at head-neck (**A**) and was 3.1 mm for lesions at body-tail (**B**).

## Data Availability

All data generated or analyzed during this study are included in this published article (and its Additional file 1).
