# Supplementary material for: Threshold of main pancreatic duct for malignancy in intraductal papillary mucinous neoplasm at head-neck and body-tail
Source: BMC Gastroenterol. 2022 Nov 19;22:473. doi: 10.1186/s12876-022-02577-3 (PMC9675150; doi:10.1186/s12876-022-02577-3)

**Threshold of main pancreatic duct for malignancy in intraductal papillary mucinous neoplasm at head-neck and body-tail**

Hao Zhou^1^, Xiaoshuang Li ^2^, Yajie Wang^1^, Zhiyue Wang^2^, Jingrong Zhu^1^, Zhongqiu Wang^1^, Xiao Chen^1^

1 Department of Radiology, Affiliated Hospital of Nanjing University of Chinese Medicine, Nanjing 210029, China

2 Department of Radiology, The Second Affiliated Hospital of Nanjing Medical University, Nanjing 210000, China

Hao Zhou and Xiaoshuang Li were contributed equally to this work

Corresponding author: Xiao Chen, Department of Radiology, Affiliated Hospital of Nanjing University of Chinese Medicine, Nanjing 210029, China, Email: chxwin@163.com or Jingrong Zhu, Department of Radiology, Affiliated Hospital of Nanjing University of Chinese Medicine, Nanjing 210029, China, Email: fsyy00597@njucm.edu.cn

**Supplemental Fig. 1** Receiver operating curve to calculate the threshold of main pancreatic duct (MPD) in identifying malignancy in branch-duct (BD)-intraductal papillary mucinous neoplasms (IPMNs). The threshold was 2.9 mm for lesions at head-neck (A) and was 3.1 mm for lesions at body-tail (B).


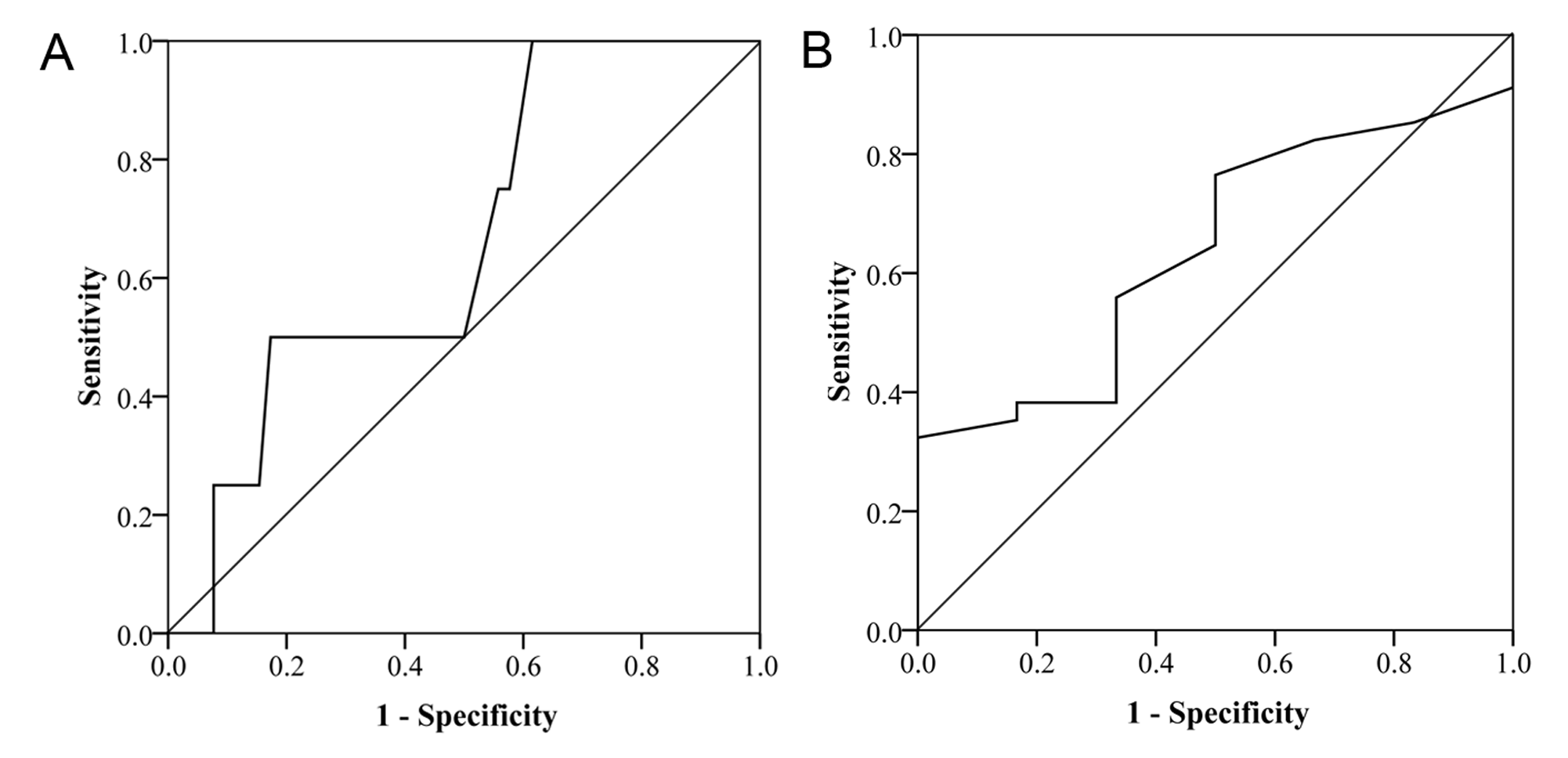

Supplement: Supplementary file 1 — Additional file 1: Fig. S1. Receiver operating curve to calculate the threshold of main pancreatic duct (MPD) in identifying malignancy in branch-duct (BD)-intraductal papillary mucinous neoplasms (IPMNs). The threshold was 2.9 mm for lesions at head-neck (A) and was 3.1 mm for lesions at body-tail (B). [file 12876_2022_2577_MOESM1_ESM.docx]
